# Feeding strategies and ageing time alter calpain system proteins activities and meat quality of Braford steers

**DOI:** 10.5713/ab.21.0227

**Published:** 2021-10-21

**Authors:** María Sumampa Coria, Dario Pighin, Gabriela Grigioni, Gustavo Adolfo Palma

**Affiliations:** 1 Animal Production and Reproduction Laboratory, NOA Institute of Bionanotechnology (INBIONATEC), Villa El Zanjón, Santiago del Estero, G4206XCP, Argentina; 2 Institute for the Agricultural Development of the Semiarid (INDEAS), Faculty of Agronomy and Agroindustry (FAyA), National University of Santiago del Estero (UNSE), Santiago del Estero, G4200ABT, Argentina; 3 National Council of Scientific and Technical Research (CONICET), Buenos Aires, C1033AAJ, Argentina; 4 Food Technology Institute - Science and Technology Institute of Sustainable Food Systems, National Institute of Agricultural Technology (INTA), Hurlingham, Buenos Aires, B1712JHB, Argentina; 5 Faculty of Agriculture and Food Sciences, Moron University, Morón, Buenos Aires, B1708JPD, Argentina

**Keywords:** Bovine, Calpain System, Calpastatin, Pasture, Supplementation, Tenderness

## Abstract

**Objective:**

The aim of this study was to evaluate the effect of ageing and feeding strategies on the calpain protease system and meat quality traits in Braford steers.

**Methods:**

Thirty Braford steers were employed; 15 animals were supplemented with corn silage during finishing and 15 were kept only on pasture. Meat quality traits and calpain system protein activity were evaluated in *longissimus thoracis et lumborum* (LTL) steaks aged for 2, 7, 14, and 21 days.

**Results:**

Aged meat showed higher pH and calcium content, while Warner Bratzler shear force (WBSF) decreased to day 21. No interaction between ageing and diet was seen for quality traits. Steers finished with corn silage showed higher values of water holding capacity, WBSF and free calcium, and lower values of pH and cooking loss. Calpain and calpastatin activities decreased with ageing. Finishing steers on pasture produced higher values of calpains and lower values of calpastatin activities. The higher values of calpain 1 activity were observed in muscles aged 2 days from pasture finished animals, and the lower activity of the inhibitor in the 21 days aged samples of the same group.

**Conclusion:**

These results suggest a diet by ageing interaction in calpains and calpastatin and this interaction impact in Warner Bratzler Shear Force in Braford LTL muscle.

## INTRODUCTION

In Argentina, beef cattle production systems have been based traditionally on pasture in temperate regions. As a result of the expansion of agriculture, beef cattle production was displaced to tropical and subtropical regions, and British breeds were replaced by other genotypes, such as composites between *Bos indicus* and *Bos taurus* breeds [1]. Numerous studies have indicated that steaks from cattle of greater *Bos indicus* genetic influence were tougher than steaks from *Bos taurus* cattle [2,3]. Nevertheless, *post mortem* strategies such as ageing could be effective for improving tenderness [4].

One of the most important proteolytic systems involved in meat tenderization is the calpain system [5]. In skeletal muscle two proteases, calpain 1 and calpain 2, as well as their inhibitor, calpastatin, have been described in detail [6]. Several studies demonstrated that calpain-1 has the most significant role in *post mortem* proteolysis and meat tenderization [7,8]. However, recent studies show that calpain-2 could contribute during extended ageing periods [4]. Giusti et al [9] suggested that lower tenderness of meat is probably not the result of a lower expression of proteases, but is more likely related to an increased calpastatin expression. This was supported by Strydom et al [10], demonstrating an increase in the value of the correlation coefficient between calpastatin activity and Warner Bratzler shear force (WBSF) within ageing time. Therefore, the aim of this study was to determine the effect of *post mortem* ageing (2, 7, 14, and 21 days) on meat tenderness and calpains (1 and 2) and calpastatin activity in beef *longissimus thoracis et lumborum* (LTL) muscle of Braford steers finished on pasture or with silage supplementation.

## MATERIALS AND METHODS

### Animals and sampling

The present study was conducted with animals from a commercial breeding herd in Santiago del Estero, in northwest Argentina (coordinates 27°17′34.3″S - 62°15′14.1″W). Animal handling and experimental procedures were in accordance with the Handbook of Procedures for Animal Welfare of the National Service of Animal Health of Argentina (Servicio Nacional de Sanidad Animal, SENASA). As was described by Coria et al [11], thirty Braford steers were randomly chosen and divided into 2 experimental groups: 15 animals were fed *ad libitum* pasture and supplemented with corn silage (1% of body weight/animal/day during 120 days) (Suppl) whereas the other 15 steers were fed *ad libitum* similar pasture without corn supplementation (Contr). Animals on both diets had free access to water. Steers, with 26±2 months of age in average, were slaughtered according to standard commercial procedures on the same day after mixing groups to avoid peri-slaughter effects. Carcasses were weighed for hot carcass weight (HCW) determination and were deboned following a 2-day cooling period at 1°C to 5°C. Samples from LTL muscle were vacuum-packed (Dise.SA., thickness 90 μm, oxygen permeability 50 cm^3^/m^2^/24 h, CO_2_ permeability 140 cm^3^/m^2^/24 h, water vapor permeability 6 to 8 g/m^2^/24 h), and stored at 2°C±1°C in darkness for the corresponding ageing time (2, 7, 14, and 21 days); subsequently they were frozen at −18°C until analysis. Before meat quality measurements, samples were thawed at 4°C±1°C overnight.

### Muscle pH and colour

Meat colour was assessed using a Commission Internationale d’Eclairage Lab System, which provides values for colour components: L* (black–white, lightness) and the chromatic coordinates a* (+ to −, from red to green) and b* (+ to −, from yellow to blue). Measurements were carried out using a Minolta CR-400 colorimeter (Konica Minolta Sensing, Inc., Bergen, NJ, USA). The instrumental conditions were artificial D65 illuminant, 8 mm port size and 2-degree standard angle observer. Determinations were carried out on 2.5 cm thick steaks. Each sample was allowed to bloom for 45 min at room temperature prior to the first measurement, and six scans of each steak were averaged for statistical analysis. Then, muscle pH was measured using a Testo 205 pH meter (Testo AG, Lenzkirch, Germany). Each measurement was performed in triplicate, taking the mean values as the assay result.

### Water holding capacity and cooking loss

The water holding capacity (WHC) was determined in duplicate following the filter paper press methodology described by Coria et al [11]. The WHC was expressed as the percentage of meat-free juice expelled (WHC = meat area/total liquid infiltrated area×100). This procedure assumes that the area of the ring of expressed juice absorbed by the filter paper is related to the amount of meat-free water.

The cooking loss (CL) was determined only once by measuring the sample weight before and after heat treatment. Post cooking measurements were done after 20 min cooling at room temperature. Steaks were subjected to heat treatment with dry heat cooking (Ingeniería gastronómica, Buenos Aires, Argentina; grill temperature, 180°C; temperature of the thermal centre of the samples, 71°C). The CL was reported as the percentage of weight loss with respect to the initial weight of the sample.

### Instrumental tenderness analysis

Instrumental tenderness was measured using the Warner-Bratzler test, assessing the resistance to a shear force in cooked meat. After cooking, samples were cooled down to 4°C overnight in refrigerator. Then, cores of 1.3 cm diameter were obtained parallel to the muscle fiber orientation and sheared once across the middle (perpendicular to the fibers) using a 1.016 mm Warner Bratzler blade (WB) probe in a TA.XT Plus Texture Analyzer (Stable Micro Systems Ltd, Surrey, UK). The test was performed with a crosshead speed of 2.0 mm/s (pre-test), 2.0 mm/s (test), 10.0 mm/s (post-test). The highest peak in Newton (maximum shear force) was measured in 7 to 10 cores per sample.

### Free calcium concentration

Muscles samples (12 g) held at −80°C were removed from frozen storage and kept at room temperature for 10 min before they were finely diced, and then kept on ice again for 20 min before they were centrifuged at 20,000×g during 20 min at 4°C. An aliquot of the supernatant was mixed with 4 M KCl. Finally, calcium concentration was determined in triplicate using the Ca-Color AA kit (Wiener Lab, Rosario, Argentina) following the manufacturer’s instructions. Absorbance was read at 570 nm in a NanoDrop 2000c UV-Vis spectrophotometer (Thermo Scientific, Barrington, IL, USA).

### Collagen content

Meat texture is determined by collagen and is improved with prolonged *post mortem* ageing [12]. Therefore, total collagen content was determined at the beginning and at the end of ageing storage. Duplicates of each sample (4 g) were hydrolyzed with sulfuric acid. A solution of hydroxyproline, made from a stock solution (600 μg/mL), was used to obtain a standard curve. Samples and standard solution were added to an oxidant solution (chloramine-T) and the formation of a reddish-purple complex with 4-dimethylaminobenzaldehyde was read at 560 nm using a NanoDrop 2000c UV-Vis Spectrophotometer (Thermo Scientific, USA).

### Calpains and calpastatin activity determination

Activity was measured in frozen samples using the method described by Coria et al [11]. Briefly, from each sample, duplicates of 200 mg of meat were homogenized in 1 mL of extraction buffer (100 mM Tris-HCl; 10 mM ethylenediaminetetraacetic acid; 0.05% β-mercaptoethanol; pH 8.3) at 13,500 rpm using a D-160 Handheld Homogenizer (Dragon Lab) and centrifuged for 30 min at 4°C at 8,800×g. An aliquot of the supernatant was mixed with glycerol at a final concentration of 30% and stored at −80°C until calpain activity was analyzed. Concentration of proteins was determined with the Quick Start Bradford protein assay (Bio-Rad, Hercules, CA, USA) following the standard micro plate protocol. For calpastatin activity, another aliquot of supernatant was heat treated in a water bath at 100°C for 5 min, centrifuged during 5 min at 20,000±g, and stored at −80°C until analysis.

Calpain and calpastatin activity was determined using the casein zymography. Clear bands indicating calpain activity were quantified comparing the density of each band with the reference standard of each gel. Before statistical analysis, calpain band densities were calculated as a percentage of a standard included in each gel. The standard was a sample of pasture-finished steers taken randomly in the same experiment.

### Statistical analysis

Data were checked for normality using Infostat Software [13]. Meat trait data were analyzed using mixed model methodology. Ageing time, feeding treatment (Suppl or Contr) and their interaction were included as fixed effects and paddock was included as a random effect in the statistical model. For meat traits, HCW was used as covariate. The choice of residual covariance structure was based on the magnitude of the Akaike Information Criterion (lowest is better). If no significant interactions were observed, the data were reanalyzed for main effects only. When a fixed effect was significant, the least significant difference Fisher test was performed to determine differences between individual treatment means. For all assays, the level of significance was set at 0.05. Linear and quadratic relationships were detected by response curves using orthogonal polynomial contrasts.

## RESULTS AND DISCUSSION

### Meat quality

There was no significant interaction (p>0.05) between the main effects of all analyzed variables. Therefore, each effect is discussed individually.

#### Ageing effect

The effect of the different ageing time on meat quality is shown in Table 1. Ageing time produced significant differences in meat pH, showing higher values after 7 days. Nevertheless, the ultimate pH values observed were within the normal range (5.6 to 5.7) [14]. This effect was described by Stanišić et al [15] and Wyrwisz et al [16]. After slaughter, the pH decreases due to the accumulation of lactic acid that occurs during anaerobic glycolysis. Then, pH increases due to the accumulation of post-slaughter (alkaline) protein degradation products, generally caused by the activity of endogenous enzymes, mainly calpains, during ageing [16].

Significant effect of ageing was observed in colour parameters lightness (L*) and yellowness (b*), even though no differences were observed in redness (a*). These results are in accordance with a study published by Stanišić et al [15], who showed a significant increase in L* and b* during the ageing of *longissimus dorsi* muscle. Higher values observed in L* parameter during ageing could be explained by the protein degradation that occurs during ageing, that leads to the weakening of protein structures, resulting in a greater scattering of light, thus increasing the lightness of meat [17]. Similarly, higher values in b* parameters were described in Brangus steers finished on pasture, in Friesian x Simmental bulls raised in semi-intensive systems, and in Angus cows and bulls finished on pasture with 14 days of ageing [16, 18,19]. Wyrwisz et al [16] found b* parameter increased during ageing and explained this difference by changes in the metmyoglobin amount due to meat surface oxidation. Marino et al [20] suggest that ageing could increase the permeability of the sarcolemma to myoglobin with easier accessibility of oxygen leading to a redder meat. Nevertheless, redder meat was not seen in the present study in LTL muscle of Braford steers aged for 21 days.

Warner-Bratzler shear force values decreased linearly with ageing time until day 21. The percentage changes were in average of 13.8%, 31.6%, and 42.4% after 7, 14, and 21 days, respectively, with respect to the initial value (day 2 *post mortem*). These results were consistent with previous publications, although the rate of decline varies. Papaleo Mazzucco et al [18] reported a 13.7% decrease of WBSF after 14 days of ageing in Brangus steers. In Angus heifers and bulls, Tullio et al [19], found a decrease in the WBSF values of 55.9% in animals finished on grass, and a decrease of 62.5% in animals finished in feedlot after 14 days of ageing. Wyrwisz et al [16] reported a reduction of 28.9% in WBSF values in Holstein-Friesian×Simmental crossbreed bulls muscle aged 21 days. Differences in WBSF values could be explained by different degree of proteolysis in muscle fibers as a result of breeds and nutritional treatment evaluated. Furthermore, improved tenderness during ageing is a result of proteolytic changes that have a major impact on tenderness development. Proteolytic enzymes cause degradation of muscle proteins, leading to changes in muscle fibers, which is correlated with a reduction in the hardness of meat [21].

The WHC and CL values remained constant during ageing time. These characteristics are pH-dependent [21]. *Post mortem* lactic acid formation reduces the ability of meat to hold water. This phenomena is related to the fact that muscle proteins being in their isoelectric point (for myosin isoelectric point = 5.4) with the weakest ability to bind water [22]. The WHC increases along with the increase in pH values of meat, due to an increase in the overall negative charge of proteins, which results in repulsion of the filaments, leaving more space for the water molecules [22]. In this study, an increase in pH during ageing was not enough to produce significant differences in WHC and CL values.

Calcium concentrations were between 0.64 mM and 0.93 mM and increased linearly during ageing. Likewise, Parrish et al [23] reported that free calcium in beef *longissimus* muscle at 10 and 14 d of ageing were 0.64 and 0.97 mM, respectively. Furthermore, Senaratne [24] documented that free calcium increased from 0.79 to 0.95 mM from 8 to 28 days of ageing. Consequently, calcium increase during early *post mortem* ageing could facilitate activation of calpains with subsequent proteolysis of muscle proteins resulting in an improvement on meat tenderness.

In the present study, no differences were observed in between 2 and 21 days of ageing (3.67±0.33 and 2.93±0.51, p = 0.055). Similarly, in other studies, the total collagen content did not differ in *semimembranous* and *longissimus dorsi* muscles of bulls evaluated at 5 and 12 days of ageing [25,26]. Total collagen content is mainly responsible for the background toughness of meat *post mortem*, which is the minimal toughness that can be reached by meat after prolonged storage [27]. As expected, 21 days of ageing were not enough to produce a collagen content decrease in LTL muscle of Braford steers.

#### Feeding treatment effects

Feeding strategy is the management factor which is most actively used in relation to improvement and/or control of animal performance, nutritional value, eating and technological quality. In agreement with Teira et al [28], corn-finished animals have lower pH value than the steers that did not receive grain at the end of the finishing period. This could be related to differences in muscle glycogen content, higher in animals fed corn silage [29]. Results of LTL muscle color determination are shown in Table 2. There were no differences in colour parameters between dietary treatments in the present study. Similar, other researchers did not find significant effects of different forage/concentrate ratio diets during finishing on muscle colour [30,31]. Faucitano et al [32] suggest that diet characteristics do not have capital relevance on meat colour, probably due to transformation processes that take place in the rumen.

No difference was found in WHC between feeding strategies. Similarly, in other studies, feeding strategy did not produce differences in WHC [32,33]. Nevertheless, the values of CL were significantly different between feeding systems, resulting in meat with lower CL in the supplemented group compared with pasture alone. Many studies have found similar differences in CL between forage- and grain-fed cattle [34,35]. Furthermore, differences observed in pH values between groups and in mechanisms involving *post mortem* proteolysis like the calpain/calpastatin system can also affect CL values [22]. Explaining the higher CL values obtained in this group [36], pasture-finished steers resulted in higher pH values and activity of the proteolytic enzymes, and thus produced higher protein fragments, which are more easily lost from the structure during storage and cooking, along with water.

Cooking loos and WHC values were higher than those obtained in other studies performed in Braford steers [30,37]. Nevertheless, in the present study meat was frozen and thawed before being measured, and previously it was reported that cell membrane can be damaged during frozen and thawed process, explaining differences with other studies [38].

As can be seen in Table 2, the calcium content was higher in meat from supplemented steers. Calpains and calpastatin proteins requires the presence of calcium to be activated [21]. In general, less calcium concentration is required for calpastatin to bind to calpains than the calcium concentration required for calpains to reach half-maximal activity, suggesting that if calpains and calpastatin were both presented in sarcoplasm, higher calcium concentrations would result in binding of calpastatin to calpains before it could initiate proteolytic activity [39]. Differences in free calcium concentration were previously associated with vitamin supplementations [40]. Interestingly, the supplementation used in those studies, increased serum calcium in early *post mortem* creating an influx of calcium into the skeletal muscle causing a decrease in calpain levels, or potentially an increase in calpastatin activity. Likewise, in the present study corn silage supplementation offered could produce the mentioned effect by increasing polyunsaturated fatty acids of sarcoplasmic reticulum membrane, making it more susceptible to oxidation and thereby causing early release of calcium [39]. Although the actual mechanism of oxidation influenced membrane degradation is unclear, Senaratne [24] showed that increased release of calcium during *post rigor* produce delayed and/or reduced proteolysis in beef, explaining the differences observed in the present study between feeding strategies in calpains and calpastatin activities and in WBSF values.

The WBSF of beef from the corn-supplemented group were higher than for beef from the pasture-only group. The higher pH values in grass-fed steers compared to grain-fed steers, influences the activity of endogenous enzyme systems at slaughter which, in turn may affect myofibril fragmentation and thus affects ultimate meat tenderness. According to Destefanis et al [41] classification, meat from supplemented animals was hard, while beef from pasture finished animals was acceptably tender. In this sense, Latimori et al [42] showed that meat for Aberdeen Angus, Charolais×Angus and Holando Argentino steers finished on pasture, with corn silage supplementation (0%, 7%, and 1%) or with concentrate can be produced without detrimental effect on meat quality, including tenderness. Furthermore, shear force values at one day of aged were similar between steaks from pasture- and concentrate-fed Hereford steers [43]. Additionally corn-finished Braford steers displayed higher WBSF values in 2 day aged meat [11]. Likewise, del Campo et al [30] did not find differences in meat from Hereford and Braford steers finished with high quality pasture compared to meat from animals finished with rangeland plus corn grain. In this sense, del Campo et al [29] suggested that when forage-finished cattle has been compared with concentrate-finished cattle at a common endpoint such as bodyweight, fat thickness over the LD, or degree of marbling, meat quality differences between diets were minimized.

In agreement with another study that has assessed the influence of diet on meat quality and collagen properties, in the present study the collagen content did not differ between the feeding groups [44]. These authors suggest that cattle feed to comparable weights and grades, type of diet had little influence on collagen content [38].

### Calpain system

There was significant interaction between ageing and feeding effect on calpain-1 and calpastatin activity. The higher values of calpain 1 activity were observed in muscles aged 2 days from pasture finished animals, and the lower activity of the inhibitor in the 21 days aged samples of the same group (Table 3).

Most literature indicates that calpain-1 activity is diminished by 14 or 21 days of *post mortem* ageing in beef [4,7]. In the present study, calpain 1 activity decreased significantly with 21 days of ageing (Table 3). Activity at day 21 remained close to 37% of its initial activity. Calpain 2 activity decreased with ageing, getting values close to 83% of its initial activity at 14 days of ageing. Similarly, other authors have described that calpain-2 activity remained close to 80.2% of its initial activity after 14 days [45]. Additionally, calpastatin activity decreased significantly with 21 days of ageing, getting values close to 67% of its initial activity (Table 3). Previous research differs from those obtained in the present study, because the inhibitor activity could only be determined during the first 48 h *post mortem* or until 5 days of ageing [46,47]. Differences observed could be explained by methods used for activity determination. According to Kristensen et al [48], calpastatin activity is often miscalculated as a result of a masking effect when it was assayed by chromatography. If calpastatin binds to inactivated calpain in the assay, the binding sites on calpastatin for active calpain molecules are masked and the calpastatin activity would be underestimated [48].

In our study, finishing steers with corn silage produced lower levels of proteases and higher levels of the inhibitor. In previous research, Volpelli et al. [49] suggested that diets had no effect on calpain and calpastatin activities. However, several authors have described that energy, vitamin supplementation and/or compensatory effect can change calpain system protein activities [40,50]. The *pre rigor* muscle environment is critical in determining the behaviour of myofibrillar proteins and their subsequent impact on meat quality attributes such as tenderness and colour. According to Dransfield [51], the effects of calpains and their inhibitors immediately *post mortem*, depend on pH and have an important influence on tenderness. Muscle pH and temperature also interact continuously during rigor development as they impact on both physical shortening and proteolytic enzyme activity [30]. As previously described, diet can influence the energy status or free calcium content, and this could impact on activities. In the present study, corn silage supplementation produces a decrease in pH values and an increment in calcium content, and this produces an increase greater of two times in the activity of the inhibitor, generating a tendency of lower calpain activity for every unit of calpastatin. In this sense, the higher calpain activity in 2 days aged beef from pasture finished animals probably caused higher proteolysis resulting in tender meat, and the higher activity of the inhibitor in supplemented steers in 2 days aged samples resulted in tougher meat.

Several studies present compelling evidence that the calpain system plays a primary role in *post mortem* meat tenderness development during ageing [5,6]. Therefore, finishing strategies that produce alterations in muscle calcium content during early *post rigor* could result in higher calpastatin activity, and may have the potential to influence calpain activity and tenderness during ageing.

## CONCLUSION

Feeding strategies and *post mortem* ageing in LTL muscle in Braford steers had a significant effect on meat quality, specifically in tenderness. The comprehensive understanding of factors is highly relevant to the development of effective ageing strategies to maximize the positive impacts of ageing. In this sense, the results obtained suggest that ageing produces significant differences up to 21 days. Furthermore, both proteases and their inhibitor activity remained until 21 days of ageing, contributing to the degradation process of muscle fibers and tenderization of meat. A feeding strategy by ageing interaction on calpain system proteins was observed. The higher values of calpain 1 activity were observed in muscles aged 2 days from pasture finished animals, and the lower activity of the inhibitor in the 21 days aged samples of the same group. Likewise, finishing steers on pasture produced lower free calcium concentration and calpastatin activity, and therefore more tender meat than corn silage supplementation. The tougher meat obtained could be due to inactivation of calpain by excessive calpastatin activity, stressing its role on meat tenderness.

## Figures and Tables

**Table 1:** Means and standard deviation of quality attributes evaluated in *longissimus thoracis et lumborum* muscle during ageing (2, 7, 14, and 21 days)

Item	Ageing				p-value	Effect^1)^	
	
	2 d	7 d	14 d	21 d		Linear	Quadratic
pH	5.60±0.14^b^	5.66±0.10^a^	5.67±0.09^a^	5.67±0.10^a^	0.025	*	NS
L* lightness	32.42±2.46^b^	33.24±2.74^ab^	33.45±2.5^ab^	34.34±2.13^a^	0.035	*	*
a* redness	16.62±1.40	16.61±1.87	17.18±2.21	17.43±1.79	0.218	NS	NS
b* yellowness	9.14±1.59^a^	8.24±1.44^b^	9.09±1.60^a^	9.31±1.42^a^	0.034	NS	*
WBSF (N)	68.16±13.92^a^	58.76±14.37^b^	46.61±13.52^c^	39.27±11.37^d^	<0.001	*	NS
WHC (%)	34.08±4.76	36.91±4.88	35.67±5.52	35.73±5.52	0.053	NS	NS
CL (%)	33.82±2.90	35.02±3.21	34.06±3.27	34.44±1.88	0.396	NS	NS
Calcium (mM)	0.64±0.04^c^	0.53±0.15^b^	0.90±0.24^a^	0.93±0.15^a^	<0.001	*	NS

pH, potential of hydrogen; NS, no significant; WBSF, Warner-Bratzler shear force; WHC, water holding capacity; CL, cooking loss.

1) Linear and quadratic response to ageing time.

a–d Values in the same row with different letters differ (p<0.05).

**Table 2:** Means and standard error values of meat quality attributes for two feeding treatments measured in thawed *longissimus thoracis et lumborum* muscle

Item	Treatment^1)^		p-value

	Contr	Suppl	
pH	5.68±0.11	5.62±0.10	0.003
Colour lightness L*	33.61±2.63	33.11±2.51	0.287
Colour redness a*	16.93±2.02	16.99±1.69	0.857
Colour yellowness b*	9.02±1.65	8.87±1.46	0.591
WBSF (N)	48.08±16.35	58.32±16.74	0.001
WHC (%)	34.57±5.49	36.62±4.55	0.052
CL (%)	35.55±2.77	33.12±2.44	<0.001
Calcium (mM)	0.68 ±0.20	0.82±0.26	0.002
Collagen (mg/g)	3.28±0.48	3.31±0.50	0.803

pH, potential of hydrogen; WBSF, Warner-Bratzler shear force; WHC, water holding capacity; CL, cooking loss.

1) Braford steers finished with: Contr, finished on pasture; Suppl, corn silage supplement at 1% of body weight per head/day in addition to *ad libitum* pasture during 120 days.

**Table 3:** Means and standard deviation of activity values of calpains and calpastatin proteins measured in the *longissimus thoracis et lumborum* muscle of Braford steers

Protein^1)^	Feeding strategies^2)^		Ageing time				SEM	Effects^3)^		
	
			2	7	14	21		A	F	A×F
CAPN1	Contr	0.42^A^	0.58	0.54	0.31	0.25	0.01	<0.001	<0.001	0.001
	Suppl	0.31^B^	0.50	0.41	0.17	0.14				
			0.54^a^	0.48^b^	0.24^c^	0.20^d^				
CAPN2	Contr	0.83^A^	0.94	0.86	0.78	0.75	0.01	<0.001	<0.001	0.262
	Suppl	0.70^B^	0.81	0.71	0.66	0.62				
			0.87^a^	0.78^b^	0.72^c^	0.69^c^				
CAST	Contr	0.87^A^	1.22	0.96	0.78	0.52	0.06	0.004	<0.001	<0.001
	Suppl	2.06^B^	2.33	2.07	1.97	1.87				
			1.78^a^	1.52^ab^	1.38^bc^	1.20^c^				

SEM, standard error of the mean.

1) Protein activity measured by casein zymography (U/g). CAPN1, calpain-1; CAPN2, calpain-2; CAST, calpastatin.

2) Braford steers finished with: Supplementation, corn silage supplement at 1% of body weight per head/day in addition to *ad libitum* pasture during 120 days; Contr, finished on pasture.

3) p-value of ageing (A), feeding (F), and A×F effects.

Feeding strategies significant differences (p-value<0.05) are expressed as different uppercase letters between rows and ageing time significant differences (p-value<0.05) are expressed as different smaller case between columns.

A,B Values are significant between feeding strategies (p<0.05).

a–d Values within a row with different letters differ (p<0.05).
